# The Emerging Vision of the *JACMP* – Part 2

**DOI:** 10.1120/jacmp.v14i3.4501

**Published:** 2013-05-06

As well as being the oldest open‐access academic journal in radiology, the *Journal of Applied Clinical Medical Physics* also employs a very special business model. The *JACMP* is free on all counts: researchers submit manuscripts for free, subscriptions are free, and there is no charge to access the Journal from the website. This is quite rare among open‐access academic journals, but the 14‐year existence of the *JACMP* proves it can be done. This can only be accomplished by keeping our costs absolutely as low as possible, while maintaining competitive and excellent publishing quality. At times, we feel like we are walking a high wire. If you are a user, you might appreciate this model and likely you realize it is very difficult to maintain, especially as the *JACMP* continues to grow. Patients also benefit, as do physicists worldwide; however, the bills need to be paid by someone. So who should pay the invoices for copyediting, layout editing, proofreading, maintenance of the system including software updates, and other costs that arrive on our doorstep quarterly? Where the money should come from is an essential question, and another of equal importance is, where should the money be best directed?

Before we address where we should find the funds to support the *JACMP*, let us consider the cost of scientific publishing. From *Nature News and Comment* (http://www.nature.com/news/open‐access‐the‐true‐cost‐of‐science‐publishing‐1.12676), we find:

“Data from the consulting firm Outsell in Burlingame, California, suggest that the science‐publishing industry generated $9.4 billion in revenue in 2011 and published around $1.8 million English‐language articles — an average revenue per article of roughly $5,000. Analysts estimate profit margins at 20%–30% for the industry, so the average cost to the publisher of producing an article is likely to be around $3,500–$4,000.”

The article continues on to explore the costs of publishing an academic science article. The range of claimed costs to publish such an article is simply astounding. Again referring to the *Nature News and Comment* article above:

“Philip Campbell, editor‐in‐chief of *Nature*, estimates his journal's internal costs at £20,000–£30,000 ($30,000–$40,000) per paper. Paul Peters, president of the Open Access Scholarly Publishing Association and chief strategy officer at the open‐access publisher Hindawi in Cairo, says that last year his group published 22,000 articles at a cost of $290 per article.”

As you can see, the scientific publishing industry is reporting costs that vary over an astonishing two orders of magnitude, with the bulk of publishing in our field close to the average stated above. The costs for these articles are paid through subscriptions and print advertising. Sometimes the academic journals return money to sponsoring societies, but this is certainly not always true. The *JACMP* publishes at a value very close to the lowest cost number reported above. It does this while maintaining a publishing quality that essentially matches that of other academic journals in radiology and radiation oncology.

So, why are many mainstream academic publications so expensive? The most obvious initial answer is that printing and mailing hardcopy journals is expensive, at about $2 per printed issue. This explains about 15%–20% of the cost differential between print and online‐only journals. Another part of the difference is profit margins for the publisher. Reliable numbers are difficult to come by, but it is estimated in the article referenced above that the profit margin for the scientific publications division of a very large academic publisher is between 40% and 50%. The ethics of this profit, which largely goes to executive salaries and investor dividends, must be critically evaluated and balanced with the need to keep costs low; this is an essential environment for open‐access to flourish and for rapid and low‐cost dissemination of clinical information to benefit patients. It is, therefore, essential for us to ask where the money should go.

Let us assume for the moment that the consensus of our users is that the present *JACMP* publication model should be maintained. This is currently the consensus of our Board of Editors. We have already accomplished the first component of our goal: low costs and excellent publishing quality. Let us now consider some of the options for funding:

- Advertising revenue from banner ads — Advertisers pay to reach the worldwide *JACMP* community. This is an excellent baseline source of revenue, but there are only so many vendors and there is only so much physical space for banner ads.
- Grants — Universities, societies or charitable foundations may cover some of the cost of open‐access journal operations. For the *JACMP*, this potential source of revenue is so far untapped. Potential societies include the American Association of Physicists in Medicine, the Canadian Organization of Medical Physicists, and the International Organization for Medical Physics.
- Contributions — It is possible to raise some revenue through directed voluntary contributions. Perhaps a check box for contribution on our annual invoice with a certain suggested contribution? This idea has not yet been explored by the *JACMP* Board of Editors.
- Advertising revenue from other sources — The *JACMP* website has crossed the threshold of 10,000 users, 20,000 visits, and 35,000 impressions per month. It may be able to attract advertising from other sources than society corporate members. Google ads are a suggested alternative source.

While keeping the *JACMP* free is very desirable, it is also difficult to do. I welcome emails from you if you have an idea in addition to those listed above. Please remember to visit one or two of our banner advertisers every time you visit the *JACMP* website. It is an easy habit to entertain and one of the surest ways to help us maintain the totally free business model of the Journal.

Michael D. Mills, PhD

Editor‐in‐Chief

